# Forehead Arteriovenous Malformation Embolization Complicated by Glue Migration into the Cavernous Sinus: A Case Report of a Rare Complication

**DOI:** 10.1055/a-2688-3865

**Published:** 2025-11-20

**Authors:** Khalifa Al Alawi, Alreem Al Khayarin, Fatma Al Habsi, Najla Al Meraikhi, Mohudoom Meera Sahib, Taimoor Al Balushi

**Affiliations:** 1 Department of Plastic and Reconstructive Surgery, Hamad Medical Cooperation, Doha, Qatar; 2 Department of Plastic and Reconstructive Surgery, Khoula Hospital, Muscat, The Sultanate of Oman; 3 College of Medicine, Qatar University, Qatar

**Keywords:** arteriovenous malformation, sclerotherapy, craniofacial tumor

## Abstract

Arteriovenous malformations (AVMs) are uncommon congenital vascular anomalies characterized by direct, high-flow connections between arteries and veins. Forehead AVMs present unique challenges due to their aesthetic considerations, risk of complications, and proximity to critical neurovascular structures. A 26-year-old male presented with a pulsatile forehead swelling present since birth, which gradually increased in size. Doppler ultrasound and magnetic resonance imaging (MRI) revealed a forehead AVM fed by branches from the superficial temporal and ophthalmic arteries, without evidence of intracranial extension. Presurgical embolization using cyanoacrylate glue achieved 90% occlusion. However, the procedure was complicated by glue migration into the cavernous sinuses, resulting in headache and dizziness. The patient was initially managed with low-molecular-weight heparin and close clinical observation. Definitive surgical resection was performed successfully 1 month later. The wound healed without complications, and no recurrence was observed during 6 months of follow-up. This case highlights the importance of a multidisciplinary approach in managing AVMs and emphasizes the need to balance embolization risks with therapeutic benefits to achieve optimal outcomes.

## Introduction

Vascular malformations, including arteriovenous malformations (AVMs), are congenital anomalies that develop when one or more vessel types form abnormally during embryogenesis. AVMs have been broadly categorized as slow-flow malformations—such as lymphatic, venous, and capillary malformations—and fast-flow malformations, including arterial malformations, AVMs, and fistulas. 1 2 3 Jackson et al proposed an alternative classification dividing these anomalies into hemangiomas, vascular malformations (fast and slow flow), and lymphatic malformations with mixed types like lymphatic venous malformations, adding further complexity. 4

AVMs are a type of fast-flow anomaly that occurs with an incidence of 1 per 100,000 person-years. They primarily affect the head and neck (47.4%) and extremities (28.5%). 5 6 These malformations arise from direct arteriovenous connections that bypass the capillary bed, forming a nidus of dysplastic vessels that lead to high-pressure venous dilation. 7 8 Although congenital, AVMs often present later in life with clinical features such as hypervascularity, bleeding, ulceration, and pain. Intracranial AVMs pose additional risks of seizures and aneurysms, and have an annual rupture rate of approximately 1%. 9 10 11 12 Schobinger's staging system categorizes AVM into four progressive stages: Quiescence, expansion, destruction, and decompensation. 9

Management is multidisciplinary and tailored to lesion characteristics and patient symptoms. Conservative measures, such as pressure garments or topical care, are often used to reduce complications in extracranial AVMs. 6 9 Due to surgical risks, unruptured intracranial AVMs are generally managed conservatively. 13 Interventional options include embolization with agents such as ethanol, N-butyl cyanoacrylate, or Onyx, often followed by surgical resection for incomplete occlusion or persistent symptoms. 6 10 A combined approach can balance symptom relief with a reduction in recurrence.

We present a case of a 26-year-old male having a forehead AVM who underwent embolization. The procedure was complicated by glue migration into the cavernous sinus, leading to delayed surgical excision and requiring anticoagulation to prevent further symptoms.

## Case

A 26-year-old Arab male with no known comorbidities or prior surgical history presented to the Plastic Surgery Department at Khoula Hospital with a pulsatile swelling located at the midline of his forehead. The patient was initially seen in our clinic at the age of 17. The lesion had been present since birth and had gradually increased in size over time. The patient reported intermittent episodes where the swelling would acutely enlarge but subsequently return to baseline. He denied any history of trauma or bleeding from the area. He also reported no associated symptoms such as pain, headache, or changes in vision. His primary concern was the cosmetic appearance of the swelling and the fact that it interfered with wearing headwear.

Clinically, the swelling was vertically oval in shape, measuring approximately 4 × 2 cm, and elevated about 1 cm above the skin surface. It extended longitudinally along the midline of the forehead, from just above the glabella to the hairline. The mass was pulsatile and exhibited both a thrill and a bruit on examination. The lesion had a bluish hue and showed no signs of active bleeding. There was no punctum and no attachment to the overlying skin. The appearance of the swelling was distinctive and disfiguring, significantly affecting the patient's facial aesthetics. Additionally, the patient was unable to properly wear his traditional headwear due to the prominence of the lesion.

**Fig. 1:**
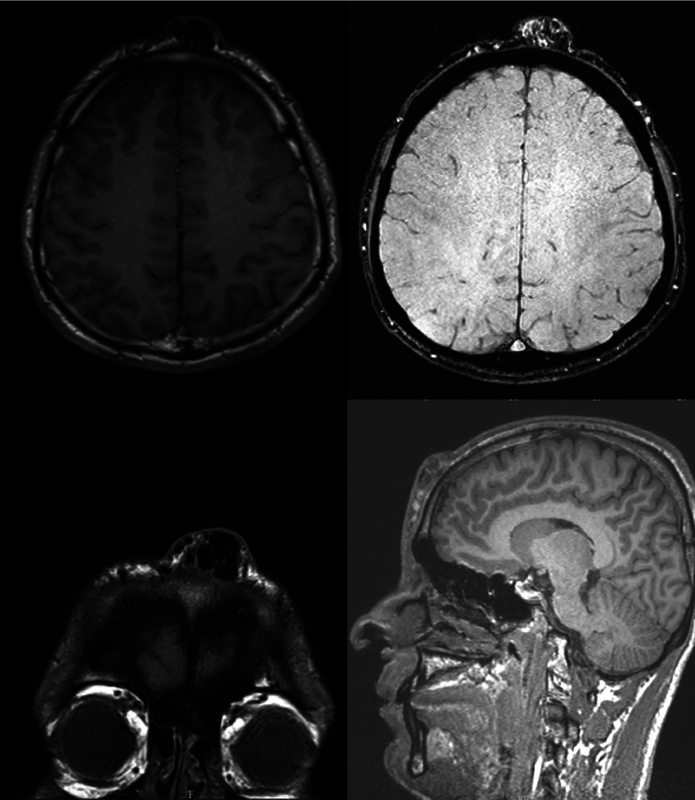
MRI reveals a high-flow extracranial arteriovenous malformation in the subcutaneous forehead tissues. The nidus is supplied by tortuous bilateral superficial temporal arteries, with a prominent midline draining vein coursing into the angular and facial veins. No intracranial extension or parenchymal abnormalities are seen. MRI, magnetic resonance imaging.

An ultrasound of the soft tissue revealed multiple tortuous, dilated vascular structures within the subcutaneous and subgaleal spaces, demonstrating a mixed arterial and venous flow pattern on Doppler study, suggestive of vascular malformations. Further assessment with magnetic resonance imaging (MRI) confirmed findings consistent with a forehead AVM supplied by the superficial temporal arteries bilaterally and drained by a large midline vein into the angular and facial veins on both sides. There was no evidence of intracranial extension ( Fig. 1 ).

The patient was reviewed at the age of 23 years and found to have a lesion that had rapidly enlarged from 4 × 2 cm to 7 × 4 cm, with an elevation of 2 cm above the skin surface within 1 year. A multidisciplinary team, including craniofacial plastic surgeons and neurointerventional radiologists, evaluated the case. In light of the lesion's rapid growth, elevation, and interference with facial aesthetics and headwear, the team agreed to proceed with presurgical embolization, followed by surgical resection the following day.

### Embolization Procedure

**Fig. 2:**
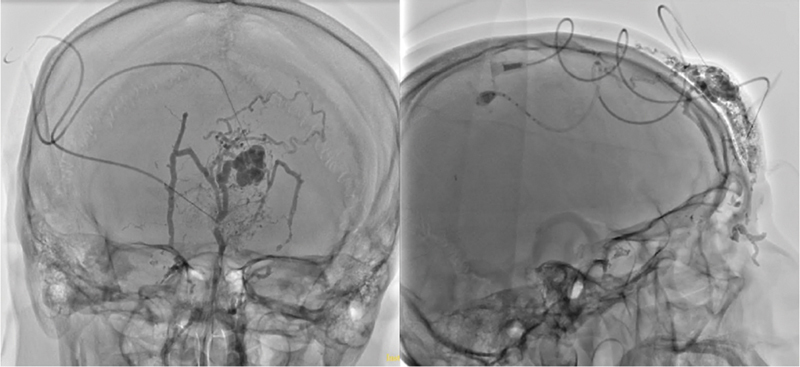
Anteroposterior (left) and lateral (right) views of intraoperative angiography demonstrating a high-flow arteriovenous malformation located in the forehead. The nidus and arterial feeders are visualized bilaterally, originating from the ophthalmic, superficial temporal, and facial arteries.

The patient underwent embolization of forehead AVM under general anesthesia. Vascular access was obtained via the right common femoral artery with a 6F sheath. A 5F STR Envoy catheter was advanced to the bilateral common carotid, internal carotid, and external carotid arteries. Angiography demonstrated a high-flow forehead AVM with feeders from the ophthalmic, superficial temporal, and facial arteries bilaterally ( Fig. 2 ). Embolization was performed using Magic Glue (cyanoacrylate), which was delivered through the left ophthalmic artery, superficial temporal artery, and percutaneous needles, achieving 90% embolization. Hemostasis was achieved using a 6F angio-seal for the right femoral artery. The patient was extubated and transferred to the high dependency unit for observation, with oxygen saturation maintained at 96%. Postprocedure care included keeping the patient supine for 5 hours, monitoring for groin hematoma, and checking right pedal pulses.

### Postprocedure Complications

**Fig. 3:**
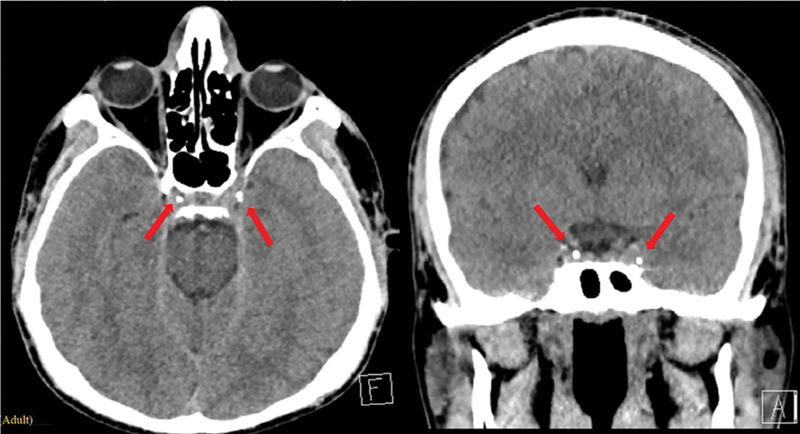
CT-scan images demonstrating glue migration into the bilateral cavernous sinuses. Glue emboli are indicated by red arrows in the images.

On the following day, the patient developed a severe headache, dizziness, and nausea, which prevented him from walking. There were no visual disturbance, ocular congestion, or groin site complications; peripheral pulses were intact. An urgent head CT was performed, which revealed a small amount of glue migration into the cavernous sinuses bilaterally, without evidence of venous thrombosis or brain parenchymal abnormalities ( Fig. 3 ). As a result, the surgical excision was postponed, and the patient was managed conservatively with antiemetics, intravenous fluids, low-molecular-weight heparin (LMWH) 4,000 IU twice daily, and close monitoring.

The patient's symptoms gradually improved. By the second day, he was able to tolerate oral intake without nausea and ambulate independently. On the fourth postprocedure day, the forehead swelling measured approximately 5 × 3 cm, with overlying dusky red discoloration and glue discharge from percutaneous needle sites. Mupirocin dressings were applied, and the patient was advised to continue local wound care after discharge. At discharge, the patient was prescribed rivaroxaban 20 mg (once daily) for 10 days. At a 2-week follow-up, the swelling showed reduced edema, with persistent skin discoloration. Importantly, there were no further neurological symptoms.

### Surgical Management and Excision

Definitive surgical excision was performed 1 month after sclerotherapy, allowing time for reduction in vascular flow and thereby minimizing the risk of intraoperative bleeding. Preoperative planning involved marking the lesion in a vertical elliptical fashion. Because the lesion was vertically oriented, excision along relaxed skin tension lines was not feasible. The marking was guided by the extent of discolored skin while ensuring preservation of enough healthy skin for primary closure.

Under general anesthesia, a skin incision was made along the premarked ellipse using a No. 15 scalpel. Dissection proceeded carefully through the subcutaneous layer using a combination of electrocautery and blunt dissection. The AVM was identified as a tangle of dilated vessels embedded within the subcutaneous tissue. Meticulous dissection was carried out to isolate and ligate feeding arteries and draining veins. Bipolar cautery and hemostatic agents such as Surgicel were used throughout the procedure to achieve effective bleeding control.

**Fig. 4:**
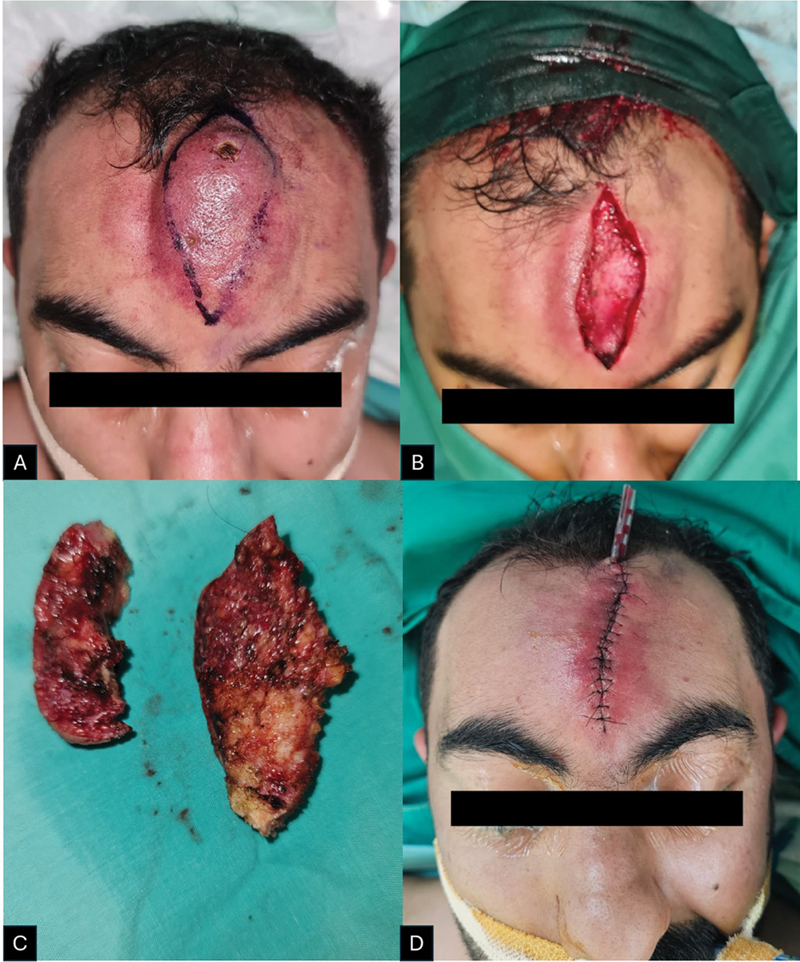
( **A** ) Forehead arteriovenous malformation (AVM) lesion with presurgical marking. The marking was designed to allow for primary closure of the defect using the skin overlying the periphery of the lesion, while ensuring excision of the skin directly over the malformation to prevent potential future skin necrosis. ( **B** ) Forehead defect following complete excision of the AVM. ( **C** ) The AVM lesion after excision. ( **D** ) The forehead defect was successfully closed primarily, without tension. A corrugated drain was left in situ.

The entire lesion, including the nidus, was completely excised with adequate margins while preserving uninvolved surrounding tissue. Closure was performed in layers: The subcutaneous tissue was approximated using 3–0 absorbable Vicryl sutures to minimize dead space, and the skin was closed with simple interrupted 5–0 Ethilon sutures to optimize the cosmetic result. A corrugated drain tube was placed, and a compressive dressing was applied to reduce postoperative swelling and the risk of hematoma formation ( Fig. 4 ).

The patient's recovery was uneventful. The drain was removed on the third postoperative day.

**Fig. 5:**
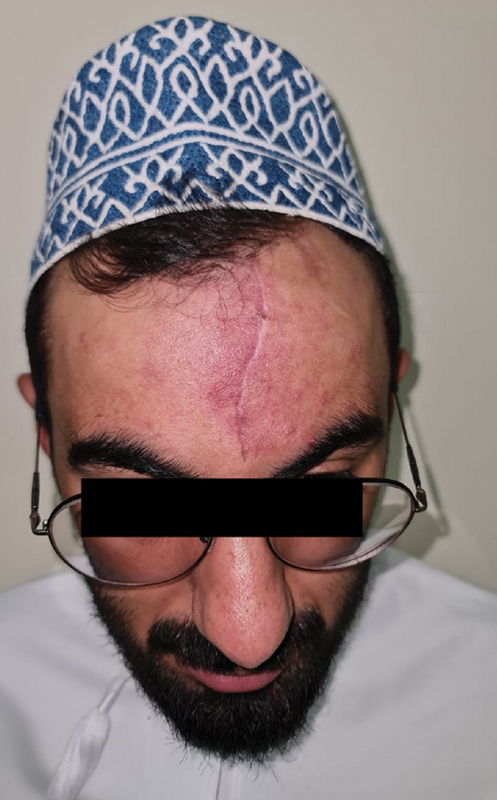
Scar at 3-week follow-up. The patient is satisfied with the outcome and is happy to wear his traditional hat, which has been lifted in the photo to allow visualization of the scar.

Follow-up visits at 1, 3, and 6 months showed a stable, well-healed scar with no signs of recurrence. The patient expressed high satisfaction with the aesthetic outcome, and the surgical site did not interfere with traditional headwear use ( Fig. 5 ).

## Discussion

AVMs are vascular anomalies characterized by complex, high-flow connections between arteries and veins that bypass the capillary network. When present in the face, these lesions pose unique challenges due to their superficial location, aesthetic implications, and the potential for severe complications if inadequately treated. 2 14

Imaging played a pivotal role in defining the extent and complexity of the AVM. Doppler ultrasound revealed mixed arterial and venous flow patterns, warranting further evaluation with MRI. In this case, the MRI confirmed arterial feeders from the superficial temporal and ophthalmic arteries, with midline venous drainage into the facial veins. The absence of intracranial extension was a favorable finding, significantly reducing the risk of life-threatening complications. This detailed imaging guided both the embolization strategy and surgical planning, minimizing procedural risks. 1

The management of AVMs can be guided by classification systems such as the Schobinger classification, which helps determine the stage and appropriate treatment approach. In general, stage I lesions are managed conservatively with close observation and regular follow-up. Stage II AVMs may be treated if they are well-localized and accessible. Stages III and IV typically require prompt intervention to prevent complications such as bleeding, ulceration, or cardiac overload. There are two primary treatment modalities for AVMs: Endovascular embolization and surgical resection. These may be used alone or in combination, depending on the lesion's size, location, and flow characteristics. 15

In our case, the multidisciplinary team opted for a staged approach involving presurgical endovascular embolization followed by surgical resection. Preoperative embolization of high-flow AVMs is widely regarded as a valuable adjunct to surgical management. 15 By selectively reducing arterial inflow to the nidus, embolization decreases the overall vascularity of the lesion. Several studies have demonstrated that preoperative embolization is associated with a significant reduction in intraoperative blood loss compared to surgical resection alone. 16 17 In addition, presurgical embolization not only minimizes the risk of excessive intraoperative blood loss but also enhances intraoperative visualization of the lesion, enabling safer and more controlled dissection. Additionally, devascularization of the AVM facilitates a more complete resection of the nidus and may allow for a smaller surgical margin, preserving adjacent healthy tissues. 16 18 Overall, this combined approach aims to improve surgical outcomes, reduce the risk of recurrence, and minimize perioperative complications.

Like other procedures, endovascular embolization of an AVM carries its own complications, including allergic reactions to the embolic material, cerebral abscess formation, hemorrhage, stroke or stroke-like symptoms, incomplete occlusion of the malformation, and migration of the glue material. 19 20 21 22 In this case, the embolization was complicated with postoperative neurological symptoms due to glue migration to the bilateral cavernous sinus, which represents a rare but serious complication. Generally, the management of glue migration following endovascular embolization can range from conservative observation in asymptomatic cases to more aggressive interventions such as endovascular retrieval or surgical removal. Symptomatic management may include anticoagulation, corticosteroids, or supportive care depending on the specific complication. 23 24 25 The choice of treatment depends on the location of the migrated glue, the patient's neurological status, and the potential risks associated with intervention. In our case, the glue migrated to the cavernous sinus, raising the risk of thrombus formation and other serious complications. Consequently, the multidisciplinary team initiated anticoagulation therapy with LMWH, transitioning to rivaroxaban at the time of discharge.

Several authors have described strategies to mitigate the risk of glue migration. These include proper planning of the injection, aided by preoperative imaging, precise catheter positioning, and controlled, slow injections under real-time fluoroscopic guidance. Additionally, altering the glue mixture to allow for controlled polymerization and using an endovascular balloon to prevent glue reflux are also recommended.” 23 25 26

Craniofacial AVMs can present significant challenges, particularly when they involve vital structures or complex aesthetic units such as the eyelids, lips, or nose. Reconstructive options range from primary closure and skin grafting to local or free flaps, or even staged excision and reconstruction. 27 28 29 30 In this case, however, the AVM was confined to the forehead region, which was a favorable factor. Since the lesion was elevated on the forehead with associated skin expansion, we determined that the optimal approach would be to reconstruct the defect using the skin overlying the peripheries of the lesion, which had already been expanded. Consequently, our surgical markings were meticulously planned to ensure there was sufficient skin for primary closure, while also excising the skin directly overlying the malformation to prevent potential skin necrosis in the future. The choice to proceed with primary closure was based on the goal of completing the procedure in a single stage, while also ensuring a superior color and texture match with the surrounding forehead skin.

### Lessons Learned

- Multidisciplinary approach is crucial: The successful management of this complex AVM case highlights the importance of involving a multidisciplinary team—plastic surgeons, neurointerventional radiologists, anesthesiologists, etc.—from the planning phase through postoperative care.
- Preoperative imaging and proper clinical assessment are essential: Comprehensive imaging (Doppler ultrasound and MRI) played a pivotal role in mapping vascular anatomy, confirming the absence of intracranial extension, and tailoring the interventional strategy. Detailed vascular mapping helps avoid surprises during embolization and surgery.
- Presurgical embolization improves surgical outcomes: Embolization significantly reduced intraoperative bleeding and improved lesion delineation. However, it introduced a rare but serious complication, which is glue migration into the cavernous sinuses.
- Early recognition and conservative management of complications can be effective: The glue migration led to neurological symptoms, but conservative treatment with anticoagulation and symptom monitoring proved effective in resolving the symptoms and preventing further serious complications. We also encourage prompt imaging for postprocedure neurological changes and anticoagulation as a viable first-line therapy in selected patients with glue migration to the cavernous sinus.

### Conclusion

This case highlights the complexities of managing forehead AVMs, emphasizing the balance between therapeutic benefits and procedural risks. The successful resolution of complications and subsequent surgical excision in this case demonstrates the efficacy of a multidisciplinary, patient-centered approach. Future advancements in embolization techniques and postoperative care protocols are likely to further improve outcomes in these challenging cases.
